# Gene-environment equivalence: The fundamental principle of Mendelian randomization

**DOI:** 10.1371/journal.pmed.1005013

**Published:** 2026-03-13

**Authors:** George Davey Smith, Gibran Hemani, Shah Ebrahim

**Affiliations:** 1 MRC Integrative Epidemiology Unit, University of Bristol, Bristol, United Kingdom; 2 Faculty of Epidemiology and Population Health, London School of Hygiene and Tropical Medicine, London, United Kingdom

## Abstract

In this Perspective, George Davey Smith and colleagues outline how and why gene-environment equivalence, the fundamental principle of Mendelian Randomization (MR), must be properly applied and critically considered in MR studies.

## Teaser

The explosion of Mendelian Randomization (MR) submissions of dubious quality to journals globally is well recognized. Contributing to this deluge of publications may be the poor understanding among practitioners, reviewers, and editors of gene-environment equivalence, the fundamental principle of MR.

**Fig 1:**
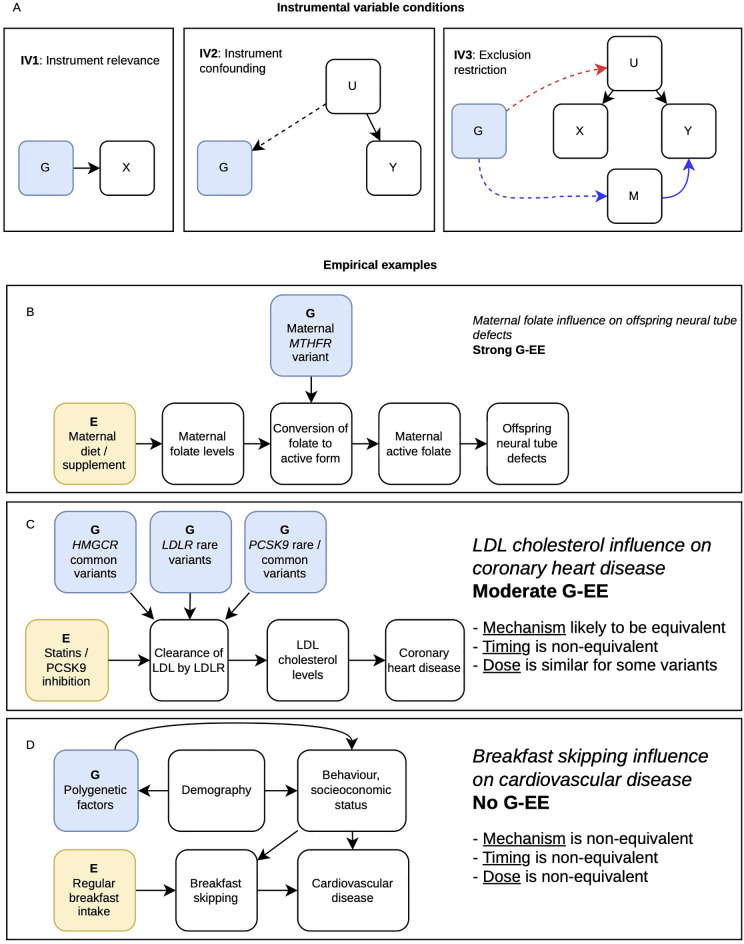
Principle of gene-environment equivalence (G-EE) and instrumental variable (IV) assumptions. **A)** The three core IV assumptions relevant to MR analyses implemented within an IV framework are that: (1) Variants (*G*) associate with the exposure (*X*); (2) there are no common causes (*U*) of the variant and the outcome (*Y*); (3) there are no paths from the variant to the outcome not mediated through the exposure (dotted lines indicate paths that violate IV conditions, e.g., for IV3 either the red or the blue paths induce violations, simple horizontal pleiotropy in blue, and correlated horizontal pleiotropy in red). **B–D)** Three examples of hypothesized causal relationships with varying degrees of how strongly the corresponding MR study design is able to recapitulate an environmental inﬂuence. B) Genetic inﬂuences on active maternal folate levels have similar timing to that of supplementation because, whilst the allele has a lifetime eﬀect on the mother, the relevant outcome in the child develops in utero. The variant has a similar magnitude of eﬀect to dietary change or supplementation. **C)** Mechanistically, the three genes—*LDLR*, *HMGCR,* and *PCSK9*—inﬂuence LDL cholesterol levels through up-regulation of functional LDL receptors (LDLR). Timing is not equivalent for the genetic perturbations—which start before birth—versus pharmaceutical interventions such as statins (*HMGCR* inhibitors) and *PCSK9* inhibitors, which generally start being taken in middle age. The effect sizes in MR studies are—as expected, given the duration of exposure differences—greater than those seen in RCTs. There are large eﬀect variants in *LDLR* and *PCSK9,* which are equivalent to pharmaceutical LDL cholesterol lowering eﬀects. G-EE is made strikingly obvious in the matching names of the genes targeted (e.g., *PCSK9*, *HMGCR*) and the pharmaceutical inhibitors of these genes [5]. **D)** Genetic variants that associate with breakfast skipping are highly nonspeciﬁc —and thus to the potential environmental intervention of promoting regular breakfasting. Some of the variants relate to other behaviors, such as smoking, meaning that there are likely many paths to CHD that are not mediated through breakfast skipping. In this case mechanism, timing, and dose do not satisfy G-EE.

Mendelian randomization (MR) uses germline genetic variants to strengthen causal inference regarding potential modiﬁable exposures for disease [1,2]. Given recent increases in low-quality submissions, publishers (including PLOS) have outlined expectations for MR papers [3,4]. These include addressing the plausibility of the instrumental variable (IV) assumptions required for eﬀect estimation for studies implemented within an IV framework [2] (see Fig 1A). We suggest that if gene-environment equivalence (G-EE)—the fundamental principle of MR—is considered critically, the potential value of an MR study is clarified.

## What is gene-environment equivalence?

The intuition behind the introduction of MR was that germline genetic variants reliably mimicking the eﬀects of an exposure can contribute to assessing the evidence for causality, strengthening the translation of observational ﬁndings into successful interventions [1]. Many of the early examples of MR involved genetic variants that modified the effect of an exposure on a disease, without actually relating to the exposure under investigation [1,6]. In such situations the variant is clearly not an IV—the first IV assumption (Fig 1A) is violated—yet provides powerful evidence regarding the causal effect of the exposure under investigation. Currently, however, the large majority of MR studies are implemented within an IV framework, in which the genetic variant reliably associates with differences in levels of an exposure. In this setting, G-EE would be seen if the eﬀect on the disease of interest generated by the genetic variants matches the eﬀect that would be seen if the same exposure diﬀerence were produced through certain environmental changes (including in lifestyle, treatment and modiﬁable endogenous factors). This is G-EE, and whilst it has been implicitly [1] or explicitly [2,6–8] discussed in the literature since the introduction of MR, it has been neglected in comparison to the IV assumptions.

G-EE has its origins in clinical, ecological, and developmental genetics [6,9,10]. For example, G-EE is assumed by the breeder’s equation, which extrapolates an estimate of the selection coefficient to make a prediction regarding trait change in the next generation [11]. G-EE can also be seen in the phenotypic similarity of the recessive genetic condition Hartnup’s syndrome and the disease pellagra: both involve characteristic skin rashes, neurological symptoms, gastrointestinal problems and an episodic course. Clinically, both would be in a differential diagnosis for a patient presenting with such symptoms. Their mechanistic overlap is that in Hartnup’s syndrome genetic variation leading to low niacin activity drives the condition; in pellagra, dietary niacin deficiency is the cause [6].

Such mechanistic overlap is the core of G-EE. Cutting across this are the dimensions of timing and magnitude of exposure. The same exposure perturbation can have diﬀerent eﬀects depending on the stage during the life course in which it occurs. During development, many irreversible changes occur, and compensation for insults—be they environmental or genetic—can be entrained. This highlights the much-neglected issue of canalization—whereby biological compensation during development attenuates the effects of genetic variation or environmental changes on a trait, potentially compromising MR interpretations [1]. Furthermore, genetic perturbations are often much smaller than those produced by, e.g., pharmaceutical interventions. Thus, predicted eﬀects from MR are generally estimated from linear extrapolation from a narrow actual range of demonstrable associations. These biological complexities are often diﬃcult to express and evaluate solely within the IV framework, and designing MR studies with G-EE in mind is important for appropriately integrating their ﬁndings within evidence triangulation [8].

## Examples of gene-environment equivalence in MR

An early MR example used variants in the *MTHFR* gene, which encodes an enzyme in folate-dependent one-carbon metabolism; maternal variants that reduce its activity alter folate-cycle intermediates (including pre-conceptually and during pregnancy) and are associated with increased neural tube defect risk in offspring [1,12]. The eﬀect of lower antenatal folate levels on NTD risk is supported by randomized controlled trials (RCTs) of supplementation starting before conception or during early pregnancy. For the conceptus, it is irrelevant whether the diﬀerence in maternal folate activity were generated by folate supplementation or by diﬀerent maternal *MTHFR* alleles; i.e., there is G-EE. As the exposure period for oﬀspring is only during their gestation, genetic variation and the intervention would aﬀect them over similar time periods (Fig 1B).

A second early example relates to cholesterol and coronary heart disease (CHD) [1]. One study reported that heterozygous carriers of rare large eﬀect variants mostly in the *LDLR* gene region (which encodes a receptor that helps clear cholesterol from the blood) had higher risk of CHD [13]; this was twice the magnitude of the eﬀects of cholesterol on CHD predicted by RCTs of statins [1]. However, while the genetic variants reported in this study are known to produce diﬀerences in cholesterol levels that can be seen from birth onwards, trials often only last ≤5 years [5]. G-EE is partial in relation to duration of exposure, generating the observed difference in effect size (Fig 1C).

## Heritable confounding and gene-environment equivalence

A common issue in MR studies and G-EE is heritable confounding, whereby genetic factors influence both an exposure and an outcome. Consider the link between C-reactive protein (CRP) and cardiometabolic disease [7]. CRP is an acute phase reactant, elevated by many causes of physiological dysregulation, e.g., cigarette smoking, obesity, atherosclerosis and infection. CRP strongly associates with CHD risk, and pharmaceutical manipulation of CRP was being considered as an intervention; however, MR studies using *cis* variants in the *CRP* gene suggested that circulating CRP levels had no causal eﬀect on CHD, discouraging the development of such drugs [2]. Genome wide association studies (GWAS), however, now identify hundreds of genetic variants inﬂuencing CRP levels, many working through obvious upstream factors, such as obesity or cigarette smoking [7]. Such genetic variants would not have eﬀects equivalent to the pharmaceutical manipulation of circulating CRP, since obesity and smoking will influence health outcomes entirely independently of their eﬀects on CRP; G-EE is clearly invalid for these variants. As GWAS studies become ever larger and identify genetic variants of very small eﬀect size working upstream of the exposure of interest, this becomes a general issue for MR [7]. In some situations, where the important heritable confounders are known, multivariable MR may be able to recover sensible estimates [7], but in other contexts such heritable confounding contributes to the tsunami of published nonsensical MR studies [8]. Consider the recent stream of papers performing MR of breakfast skipping as the exposure and a vast array of outcomes. It is not plausible that a genetic variant can directly inﬂuence breakfast skipping as its principal eﬀect; rather, breakfast skipping will be downstream of a series of processes that will likely have eﬀects through other pathways (Fig 1D). In such circumstances, G-EE is very unlikely to hold.

## The plausibility of gene-environment equivalence

The *STROBE-MR* reporting guidelines [14] state that G-EE should be justiﬁed in any MR study. This is clearly not a binary situation, in which there is either complete equivalence or none at all; there will be degrees of reasonableness of the principle. Evaluation should consider the mechanisms through which germline genetic variants inﬂuence the exposure, timing over the life course of the exposure, potential dose-response relationships, and cell type and tissue location of genetic eﬀects. Reviewers and editors should consider this evaluation when deciding whether a paper deserves consideration for publication.

The emphasis on “certain” environmental modiﬁcations in our introduction of G-EE above indicates that the range of modifications being considered for each question needs to be carefully considered. For example, reduced levels of adiposity can be caused by many factors, including infections, cancers, endocrine disorders, cigarette smoking, alcoholism, severe anorexia nervosa, depression or environmental poisons. For many of these examples, genetic proxies exist, but these would not be equivalent to any actual or hypothetical interventions that would be implemented to reduce obesity levels and their consequent health burdens.

In the context of many contemporary MR studies, the notion of there being specific genetic influences on many of the exposures examined is simply implausible [8]. If attempts to construct G-EE stories lead to researcher reflexivity it could go a long way to stemming the tide of noncontributory (and often paper-mill produced) MR publications [3,4,8], and free up the time of scientists, reviewers, and editors to focus on the issues to which MR could be contributory. The reconnection with biology, and moving on from mindless application of IV analysis, could return MR to being a useful contributor to medical knowledge [8].

## Abbreviations

CHD: coronary heart disease
CRP: C-reactive protein
G-EE: gene-environment equivalence
GWAS: genome wide association studies
IV: instrumental variable
LDLR: LDL receptor
MR: Mendelian Randomization
RCTs: randomized controlled trials
